# Synthesis and characterization of ZrO_2_–ZnO heterojunction composite for isopropanol detection

**DOI:** 10.1039/d3ra06701g

**Published:** 2024-01-18

**Authors:** Hang Liu, Shenghui Li, Lvqing Wang, Shengjue Yang, Yuhong Zhang

**Affiliations:** a School of Electrical and Computer Engineering, Jilin Jianzhu University Changchun 130118 China zhangyuhong@jlju.edu.cn

## Abstract

We prepared ZrO_2_–ZnO heterojunction composites by a simple hydrothermal method as materials sensitive to isopropanol gas. The 5% ZrO_2_–ZnO sample presented a uniform rod-like structure. The optimum operating temperature, sensitivity and response/recovery times were measured to investigate the response of ZrO_2_–ZnO composites to isopropanol. The sensor based on 5% ZrO_2_–ZnO composites at an optimum temperature of 260 °C had a response to 100 ppm isopropanol of up to 172.46, which was about 3.6 times higher than that of pure ZnO. The sensor also exhibited fast response and recovery times of 5 s and 11 s, respectively. The gas-sensitive properties can be attributed to the rod-like structure, heterojunction structure and catalytic activity of ZrO_2_. These results would contribute in expanding the application of ZrO_2_ in gas sensors.

## Abstract 2

We prepared ZrO_2_–ZnO heterojunction composites by a simple hydrothermal method as materials sensitive to isopropanol gas. The 5% ZrO_2_–ZnO sample presented a uniform rod-like structure.

## Graphical abstract

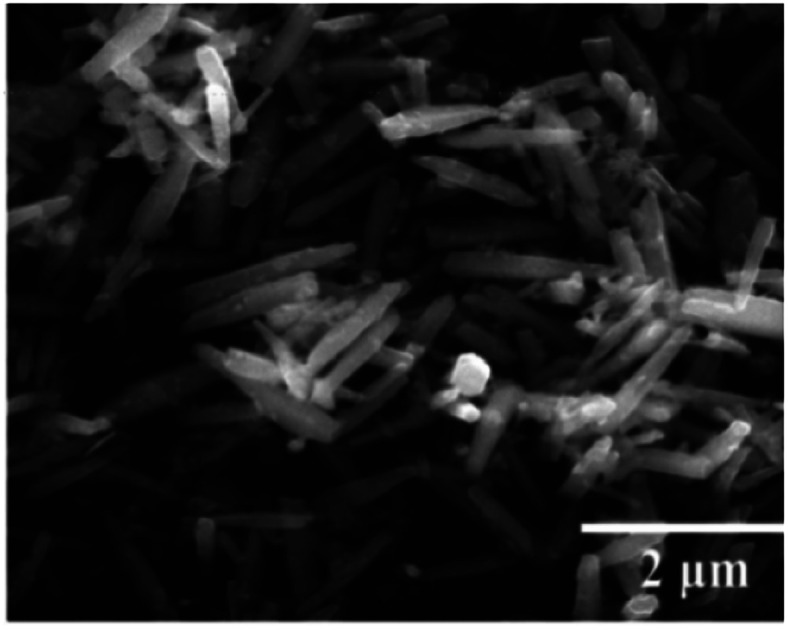

## 1. Introduction

Isopropanol belongs to the category of volatile organic compounds(VOC), which is an organic, colorless, and flammable liquid.^1^ It is commonly used as a solvent in paint, cosmetic and pharmaceutical industries^2^ and as a detergent to remove oil, grease and other treatment dirt from printed circuit boards.^3,4^ However, isopropanol is a risk to human health and will slightly irritate the airways at concentrations >400 ppm.^5^ If people are exposed to an environment with high concentrations of isopropanol, they may experience dizziness, lung damage and even coma.^6,7^ The World Health Organization's International Agency for Research on Cancer published a preliminary compilation of references to their list of carcinogens in 2017. Isopropanol was affirmed as one of the three carcinogens that can be used for early diagnosis of lung cancer by detecting changes in their concentrations in exhaled breath.^8,9^ The accurate detection of isopropanol will therefore contribute to protecting life.^10^ Therefore, a series of isopropanol sensors are designed, such as gas chromatography and spectroscopy, which are expensive and exhibit low portability;^11^ the practice applications are limited. The metal oxide semiconductor (MOS) gas sensors attracts much interesting because of their lower power, high sensitivity, small volume and easy integration.^12^ Hu *et al.* have synthesized SnO_2_ nanorods through the chemical precipitation method, and the response of sensor to 100 ppm isopropanol is about 17.^13^ Cai *et al.* have prepared ZnO–CdO composites with a response of about 3.5 to 100 ppm isopropanol.^14^ Thus, designing high sensitivity isopropanol sensor based on MOS materials is an important research field.

It is well known that common MOS materials, such as In_2_O_3_, SnO_2_, WO_3_, NiO, Fe_2_O_3_ and ZnO, have been used as gas-sensitive materials.^15–18^ Among them, ZnO is an n-type semiconductor with a 3.7 eV band gap. According to previous reports, ZnO-based gas sensors have the advantages of a high response, short response-recovery time and good selectivity for the detection of toxic and combustible gases.^19–22^ However, the gas-sensitive performance of ZnO-based sensors is still far from satisfactory in industrial settings.^23^ Some strategies have been proposed to improve the gas-sensitive properties of semiconductor materials, such as enhancing the specific surface, doping with noble metals and designing heterostructures.^24,25^

ZrO_2_, with a wide bandgap of 5.0–5.85 eV, has unique catalytic activities.^26,27^ ZrO_2_ possesses four surface properties: acidic, basic, oxidizing and reducing. A series of binary oxide semiconductors, such as (ZrO_2_–SnO_2_, ZrO_2_–In_2_O_3_, ZrO_2_–TiO_2_ and ZrO_2_–Al_2_O_3_) have been designed for their catalytic activity.^26–29^ In recent years, binary oxide semiconductors based on ZrO_2_ and other MOSs have been applied to gas sensing.

Song *et al.* synthesized In_2_O_3_–ZrO_2_ nanowires *via* a facile electrospinning strategy. The In_2_O_3_–ZrO_2_ heterojunction showed a fast response to 100 ppm acetone gas with a response time of ∼1 s.^26^ Jin *et al.* reported a solution combustion technique to synthesize coral-like macro-/mesoporous ZnO–ZrO_2_ composites for the detection of isopropanol.^7^ Shen *et al.* reported the development of ZrO_2_-doped ZnO gas-sensitive films *via* an inkjet printing method; the 3% ZrO_2_–ZnO film showed the highest sensitivity to 180 ppm acetone, about 2.3 times that of the pure ZnO film.^30^ Li *et al.* prepared ZrO_2_–ZnO nanocomposites *via* electrostatic spinning. The sensors showed a response of 107 to 100 ppm butanol.^31^ Wang *et al.* prepared ZrO_2_ and ZnO hybrid nanocomposites for designing electrochemical sensors.^32^ The sensor was highly selective and ultrasensitive for epinephrine, uric acid and folic acid. However, few studies have been conducted to prepare ZrO_2_–ZnO composites *via* a facile hydrothermal route for the detection of isopropanol.

We synthesized ZrO_2_–ZnO heterojunction materials by a simple hydrothermal method and investigated their response to isopropanol. The gas-sensitive mechanisms are discussed based on the catalytic activity of ZrO_2_, ZrO_2_–ZnO heterojunction structure and specific surface enhancement of ZnO.

## 2. Experimental

### 2.1 Material preparation

**Fig. 1:**
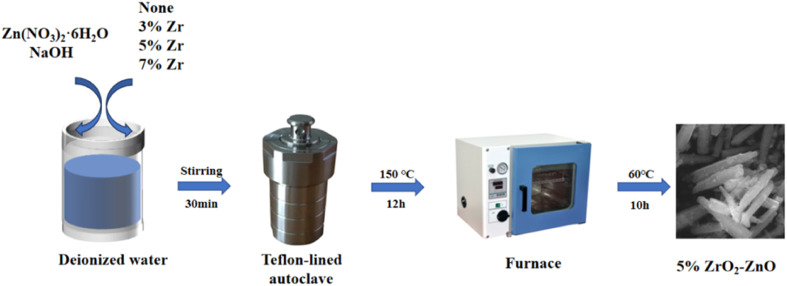
Flow diagram of the preparation method for ZnO-based sensor materials.

The raw materials included zinc nitrate hexahydrate (Zn(NO_3_)_2_·6H_2_O, 99.99%, Sinopharm Chemical Reagent Co), zirconium nitrate pentahydrate (Zr(NO_3_)_4_·5H_2_O, 99.99%, Tianjin Comio Chemical Reagent Co.) and sodium hydroxide (NaOH, 99.9%, Tianjin Fuchen Chemical Reagent Factory Co). Pristine ZnO and ZrO_2_–ZnO materials were synthesized *via* a hydrothermal method. The synthetic route of the samples is shown in Fig. 1. First, 1.8 mmol Zn(NO_3_)_2_·6H_2_O(0.535 g), *x* g Zr(NO_3_)_4_·5H_2_O (*x* = 0.007 g, 0.023 g, 0.038 g, 0.085 g) and 4 mmol NaOH (0.16 g) were put into 40 mL of deionized water. The precursor solution was obtained after magnetic stirring for about 30 min. Then, the solution was transferred to a 50 mL Teflon-lined autoclave, sealed and heated to 150 °C for 12 h. After the hydrothermal reaction was complete, the reactant was removed from the autoclave and cooled naturally to room temperature. The precipitate was filtered and washed three times with deionized water and anhydrous ethanol and finally dried in an oven at 60 °C for 10 h. At last, the samples were placed in a muffle furnace and calcined to 400 °C at 2 °C min^−1^ for 2 h. Pristine ZnO and *x*% ZrO_2_–ZnO (*x* = 3, 5 and 7) nanocomposites were thus obtained.

### 2.2 Characterization of materials

The crystalline structures of the samples were analyzed by X-ray diffraction (XRD, Rigaku Ultima IV X-ray diffractometer) with Cu Kα radiation (*λ* = 1.541 Å; 40 kV, 40 mA). The morphology of the prepared samples was characterized by field-emission scanning electron microscopy (SEM, FEI QUANTA FEG 450), transmission electron microscopy (TEM, JEM-F200) and high-resolution transmission electron microscopy (HRTEM, JED-2300T) with selected-area electron diffraction (SAED). The relative chemical compositions and distributions of different elements were evaluated by X-ray photoelectron spectroscopy (XPS, Thermo Scientific K-Alpha spectrometer, Al Kα source, 1486.6 eV) and X-ray energy spectrometry (Oxford X-MAX50). The gas-sensitive properties of the samples were measured with a chemical gas sensor-8 type intelligent gas analysis system (CGS-8, SINO AGGTECH).

### 2.3 Sensor manufacturing characteristics

**Fig. 2:**
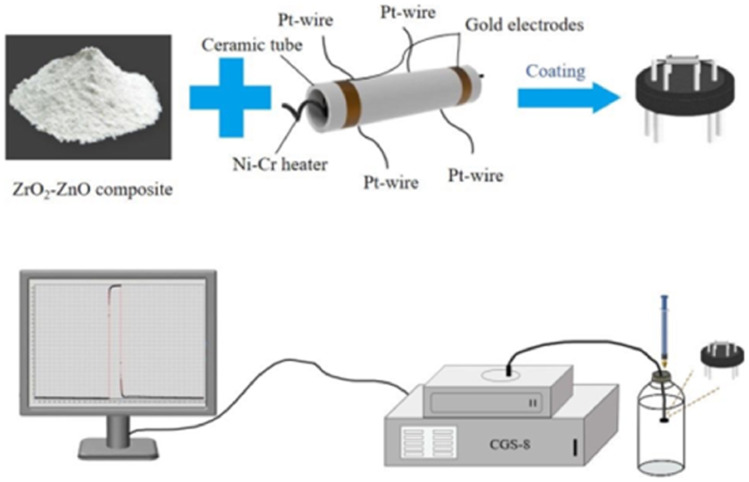
Schematic diagram of sensor fabrication and gas testing.

Fig. 2 shows the composition of the gas sensor and test procedure. The gas sensor consists of a pair of ceramic tubes with gold electrodes. The layer of sensitive material is put on the surface of the ceramic tubes. The nichrome alloy coil heater is used for temperature regulation. An appropriate amount of sample is mixed with a 9 : 1 mixture of deionized water and anhydrous ethanol to form a paste, which is then coated on the ceramic tube. After drying, the ceramic tube with the sensing material is placed in a muffle furnace and annealed at 400 °C for 2 h to obtain a gas sensor. The sensor is aged in air at 200 °C for 7 days. The sensing response is defined as the relative change in the resistance of the sensing material in air and gas: *S* = *R*_g_/*R*_a_, where *R*_a_ and *R*_g_ are the resistance values of the sensor in air and in the target gas, respectively. The process of gas testing is shown in Fig. 2. First, the corresponding concentration of gas is pumped into a glass bottle using a syringe. The sensor is then quickly put into the glass bottle and the response is observed.

## 3. Result and discussion

### 3.1 Structure and morphology

**Fig. 3:**
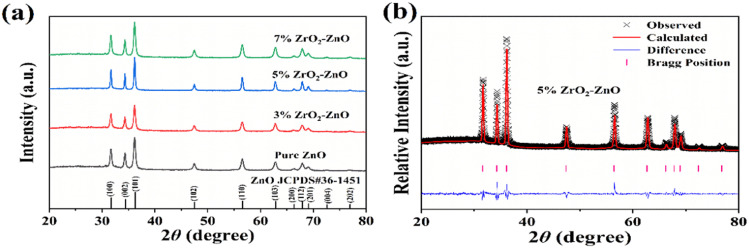
(a) XRD patterns of the pure ZnO and *x*% ZrO_2_–ZnO (*x* = 3, 5, 7) samples. (b) Rietveld plots with 5% ZrO_2_–ZnO.

The XRD patterns of pure ZnO and ZrO_2_–ZnO nanocomposites are shown in Fig. 3(a). The phase composition and lattice constants of the samples were analyzed through the XRD data. As seen in Fig. 3(a), pure ZnO and ZrO_2_–ZnO nanocomposites showed clear diffraction peaks at 31.8°, 34.4°, 36.2°, 47.5°, 56.6°, 62.9°, 66.4°, 67.9°, 69.1°, 72.5°, and 77.0°, corresponding to (100), (002), (101), (102), (110), (103), (200), (112), (201), (004) and (202) crystal planes. According to the standard card JCPDS no. 36-1451, all diffraction peaks correspond to hexagonal fibrillated ZnO. No additional peak appears in the XRD spectrum, which indicates that ZnO and ZrO_2_–ZnO nanocomposites are pure phase. In addition, no diffraction peak of ZrO_2_ is observed in the XRD patterns, which may be due to the better dispersion of elemental Zr in ZnO or low doping concentration of ZrO_2_.

XRD Rietveld refinement of the 5% ZrO_2_–ZnO sample was performed using the visualized electronic structure analysis (VESTA) procedure. The calculated patterns and Bragg position difference plots are shown in Fig. 3(b). In addition, pure ZnO and 3% and 7% ZrO_2_–ZnO were also calculated. The lattice parameters and feasibility factors (*χ*^2^, GOF and *R*_wp_/%) are given in Table 1. The results of feasibility factors satisfied the reasonable value interval, and therefore, the calculated lattice parameters were reliable. The lattice volumes of pure ZnO and ZrO_2_–ZnO samples were very similar, and therefore, the Zr^4+^ ions do not substitute at the location of Zn^2+^. ZrO_2_ is distributed on the surface of ZnO, and the ZrO_2_–ZnO junction forms a p–n heterostructure.

**Table 1:** Refinement lattice parameters of pure ZnO and *x*% ZrO_2_–ZnO (*x* = 3, 5, 7) samples

Sample	*a*, Å	*b*, Å	*c*, Å	*V*, Å^3^	*α* = *β*	*γ*	*Z*	*χ* ^2^	GOF	*R*_wp_, %
ZnO	3.2516	3.2516	5.210040	47.706	90°	120°	2	1.65	1.29	6.381
3% ZrO_2_–ZnO	3.2480	3.2480	5.2040	47.544	90°	120°	2	1.50	1.23	6.169
5% ZrO_2_–ZnO	3.25178	3.25178	5.21096	47.7190	90°	120°	2	2.28	1.51	7.563
7% ZrO_2_–ZnO	3.2454	3.2454	5.1988	47.420	90°	120°	2	0.98	0.99	7.236

**Fig. 4:**
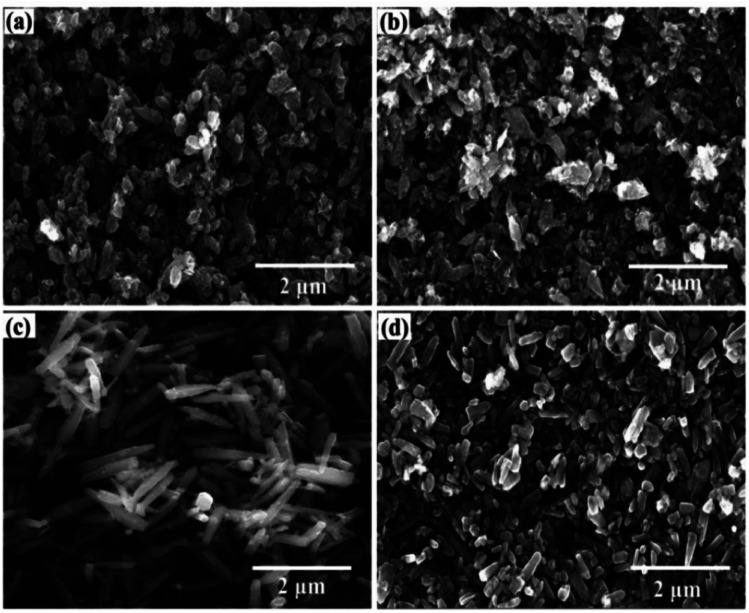
(a) SEM images of pure ZnO, (b) 3% ZrO_2_–ZnO_2_, (c) 5% ZrO_2_–ZnO_2_, and (d) 7% ZrO_2_–ZnO_2_.

The morphologies of ZnO, 3% ZrO_2_–ZnO, 5% ZrO_2_–ZnO and 7% ZrO_2_–ZnO are shown in Fig. 4. The particle morphology of pure ZnO shows a non-uniform sheet-like structure. The particle morphology of ZrO_2_–ZnO is influenced by the dopant concentration of ZrO_2_. The particle morphology of the sample is very similar to that of ZnO. However, the particle morphology of the 5% ZrO_2_–ZnO sample presents a uniform elongated rod-like structure. The length of a single rod reaches about 1.2 μm. Once the dopant concentration of ZrO_2_ reaches 7%, the rod-like structure still exists, but the length of a single rod is short. Therefore, ZrO_2_ acts as a catalyst in the ZrO_2_–ZnO system and affects the growth of ZnO.^7^

Fig. S2 shows the EDS of the 5% ZrO_2_–ZnO sample. It can be seen that the sample is composed of three elements (Zn, Zr and O), which further indicates that ZrO_2_ was successfully doped into ZnO with a uniform distribution.

**Fig. 5:**
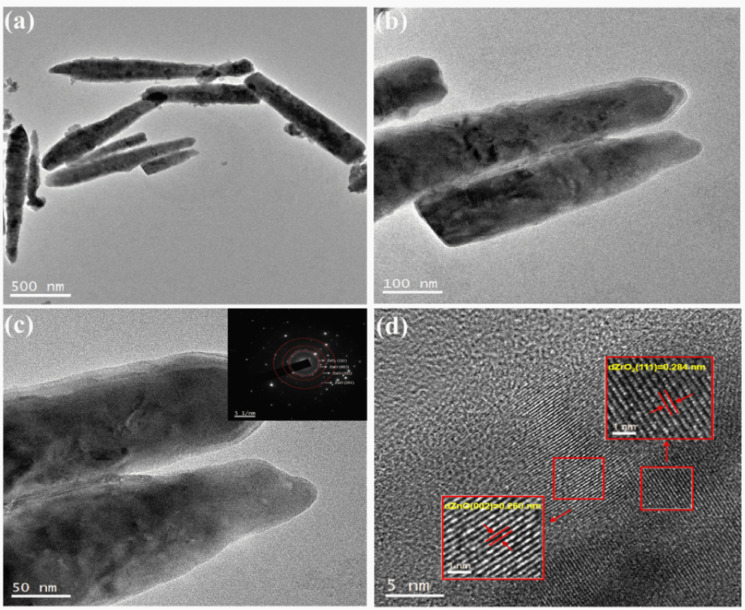
TEM and HRTEM images of 5% ZrO_2_–ZnO. Insets are the corresponding selected area electron diffraction (SAED) patterns.

The morphology and structure of the 5% ZrO_2_–ZnO complexes were investigated using the TEM images to further understand the morphology of the ZrO_2_–ZnO sample. The composite consists of nanorods about 1.2 μm long (Fig. 5), in agreement with the SEM results. The SAED map (Fig. 5(c), inset) shows that composite oxides have a polycrystalline structure with ring diffraction spots corresponding to ZnO and ZrO_2_. The high-resolution TEM image in Fig. 5(d) shows lattice stripes with an adjacent spacing of 0.260 nm, which correspond to the (002) facet of fibrillated ZnO, and lattice stripes with an adjacent spacing of 0.284 nm, which may correspond to the (100) face of Zr.^33,34^ This indicates that ZnO and ZrO_2_ are tightly connected in the composite. Only two lattice spacings and no other crystalline phase are seen, indicating that the composite oxide is well crystallized.

**Fig. 6:**
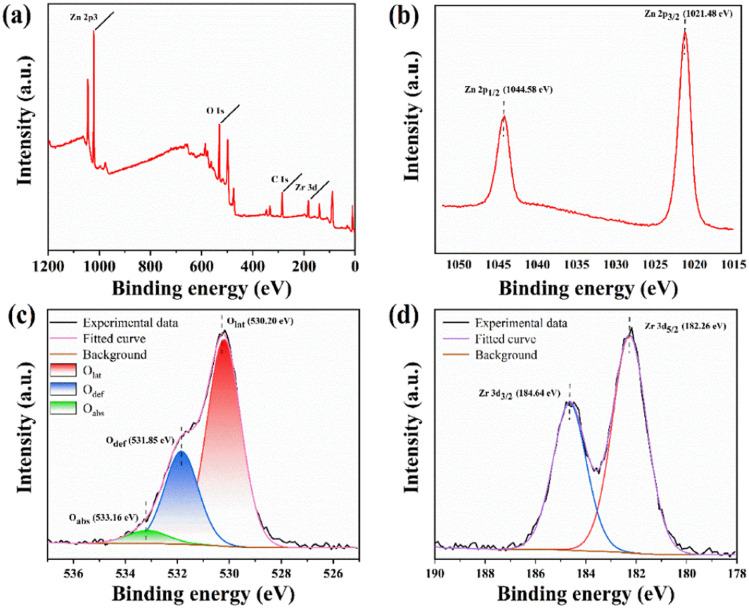
(a) XPS survey spectrum and detailed spectra of (b) Zn 2p, (c) O 1s and (d) Zr 3d for 5% ZrO_2_–ZnO.

To further analyze the elemental composition and chemical morphology of ZrO_2_–ZnO, the XPS of 5% ZrO_2_–ZnO was measured (Fig. 6). The Cls peak at 284.8 eV is used as a reference to correct all binding energy positions. Zn, O and Zr peaks were obtained, with no other impurities (Fig. 6(a)). The Zn 2p_3/2_ peak at 1021.9 eV and the Zn 2p_1/2_ peak at 1044.9 eV can be observed in the detailed spectra of Zn (Fig. 6(b)), which proves the presence of Zn in the Zn^2+^ state in the 5% ZrO_2_–ZnO composite.^35–37^Fig. 6(c) shows lattice oxygen (O_lat_, 530.20 eV), oxygen vacancies (O_def_, 531.85 eV) and chemisorbed oxygen species (O_abs_, 533.16 eV) in the detailed spectra of O.^37–39^Fig. 6(d) clearly shows Zr 3d_5/2_ and Zr 3d_3/2_ peaks at 182.26 and 184.64 eV, respectively. The difference between the two peaks is about 2.38 eV, which is in good agreement with the literature values for the Zr^4+^ state in ZrO_2_.^40,41^

### 3.2 Gas sensing characteristics

**Fig. 7:**
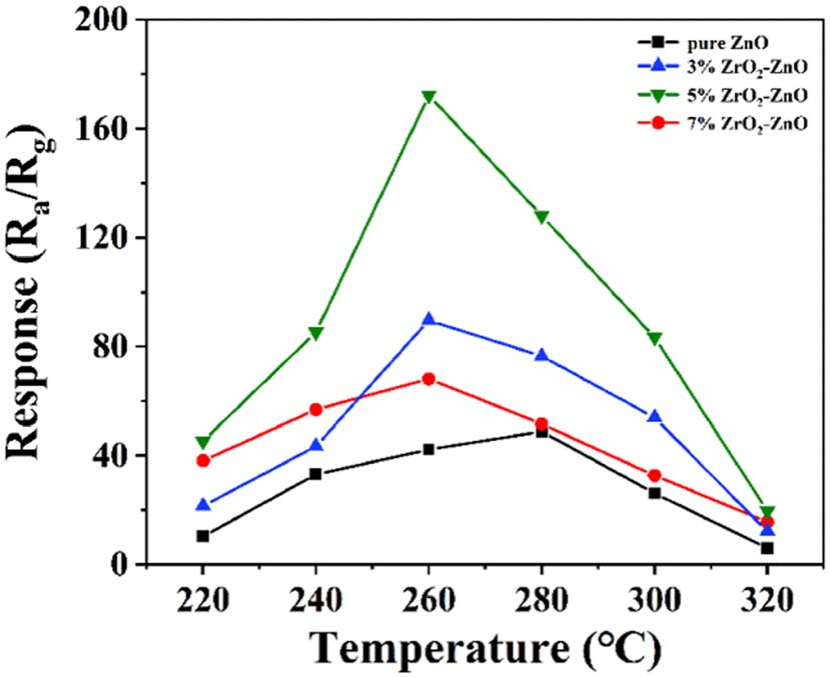
Response of ZnO doped with different amounts of Zr to 100 ppm isopropanol at different operating temperatures.

We investigated the gas-sensitive properties of pure ZnO and ZrO_2_ZnO samples for isopropanol in detail. First, the response values of ZnO and *x*% ZrO_2_–ZnO (*x* = 3, 5, 7) nanocomposites to 100 ppm isopropanol were measured at 240–340 °C (Fig. 7). The responses of all sensors present a pyramidal pattern with increasing operating temperature. It is well known that more energy is available to activate the reaction between the isopropanol molecule and adsorbed oxygen with increasing temperature. The gas response of the sensor therefore increases with temperature. However, desorption of surface oxygen species will occur when the operating temperature exceeds a certain value, which hinders the interaction between the adsorbed oxygen and isopropanol. The gas response therefore decreases at higher temperatures.

The pure ZnO sensor exhibits the maximum response of 48.89 to 100 ppm isopropanol at an operating temperature of 280 °C. The sensors with 3% and 7% ZrO_2_–ZnO composites present a much higher response to 100 ppm isopropanol at 260 °C (89.68 and 68.12, respectively). The sensor with the 5% ZrO_2_–ZnO composite has the highest response of 172.34 at 260 °C. The response is enhanced 3.53 times compared with pure ZnO, and the operating temperature decreases by 20 °C. The results may be explained by three reasons: (1) the 5% ZrO_2_–5% ZrO_2_–ZnO composite with a uniform rod structure exhibits a higher specific surface area than the pure ZnO composite, which produces more adsorbed oxygen and increases the gas response; (2) ZrO_2_ plays a catalytic role in promoting gas-sensitive reactions; and (3) the ZrO_2_–ZnO heterojunction structure also plays an important role in enhancing the gas response.^42,43^ In addition, the responses of all ZrO_2_–ZnO sensors over the whole temperature range are higher than those of pure ZnO sensors, which indicates that the formed ZrO_2_–ZnO heterojunction is beneficial to the response of ZnO sensors to isopropanol, and the content of ZrO_2_ in ZrO_2_–ZnO affects the response.

**Fig. 8:**
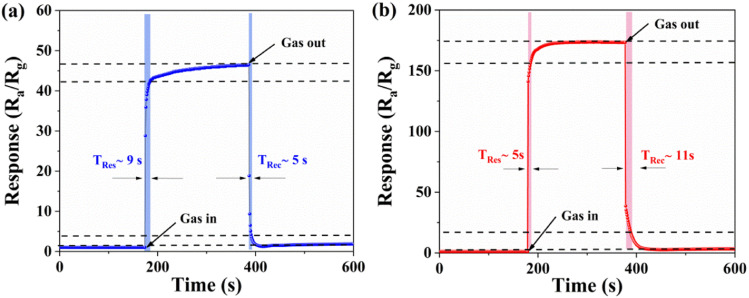
Dynamic response-recovery curves of the (a) pure ZnO and (b) 5% ZrO_2_–ZnO sensors to 100 ppm isopropanol at 280 °C and 260 °C.

The response and recovery times are also key parameters of gas sensors. Fig. 8(a) and (b) show the response-recovery curves of pure ZnO and 5% ZrO_2_–ZnO in a 100 ppm isopropanol atmosphere at 280 °C and 260 °C, respectively. It can be seen that the response and recovery times of pure ZnO are 9 s and 5 s, respectively, while those of 5% ZrO_2_–ZnO are 5 s and 11 s, respectively. This indicates that the response time of 5% ZrO_2_–ZnO is less than that of pure ZnO. The result also proves that the codoped ZrO_2_ material enhances the isopropanol response rate. The catalytic effect of ZrO_2_, uniform rod structure and ZrO_2_–ZnO heterojunction also contribute to a decrease in the response time.

**Fig. 9:**
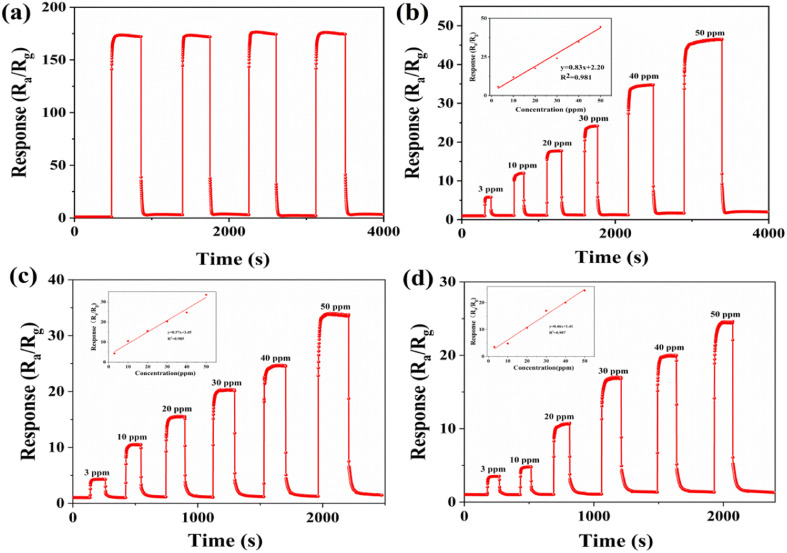
(a) Dynamic response-recovery curves of the 5% ZrO_2_–ZnO sensor for a 100 ppm concentration of isopropanol gas. Dynamic response-recovery curves of the (b) 5%, (c) 3% and 7% ZrO_2_–ZnO sensors for different concentrations of isopropanol gas at the optimum operating temperature.

Repeatability is an important performance parameter for gas sensors. The sensor with the 5% ZrO_2_–ZnO composite was tested several times with 100 ppm isopropanol at an optimum operating temperature of 260 °C. As is shown in Fig. 9(a), the response to isopropanol was almost equal over four cycles, which indicates the good repeatability of the sensor. Each time the sensor returned to the initial value. This will benefit the practical applications of the gas sensor.

The dynamic response and recovery curves of the 5% ZrO_2_–ZnO sensor for different concentrations of isopropanol (3–50 ppm) were investigated at 260 °C (Fig. 9(b)). The response of the 5% ZrO_2_–ZnO sensor reached 5.78 for 3 ppm isopropanol, which indicates that the isopropanol sensor has low detection limit and high sensitivity. The linear relationship between the response values and isopropanol concentrations was fitted (Fig. 9(b)). The least-squares conforming equation was *Y* = 2.20 + 0.83*X* (*R*^2^ = 0.981). This result shows that a good linear relationship was obtained in the concentration range 3–50 ppm isopropanol. In addition, the 3% and 7% ZrO_2_ZnO sensor responses for different concentrations of isopropanol were investigated (Fig. 9(c) and (d)). The least-squares conforming of the 3% ZrO_2_–ZnO sensor was *Y* = 3.45 + 0.57*X* (*R*^2^ = 0.985) and that of the 7% ZrO_2_–ZnO sensor was *Y* = 0.46*X* + 1.41 (*R*^2^ = 0.987). This indicates that all the sensors had a relatively good linearity.

**Fig. 10:**
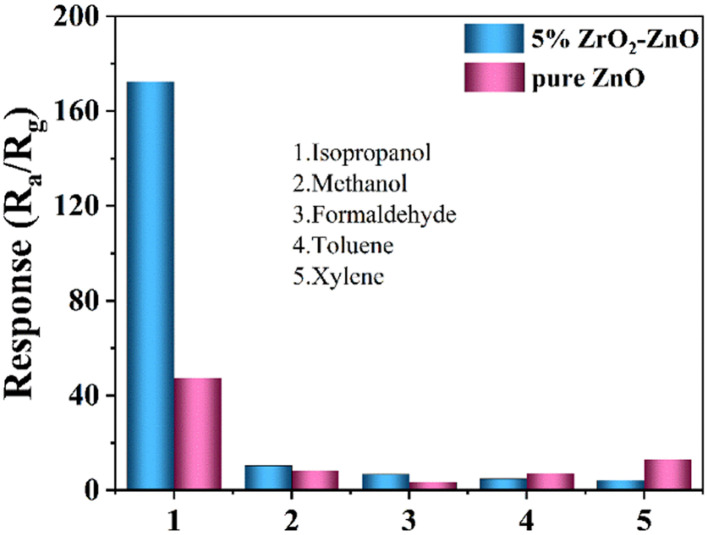
Selectivity of the 5% ZrO_2_–ZnO sensor to 100 ppm isopropanol, methanol, formaldehyde, toluene, and xylene.

Selectivity is an important parameter of gas sensors. To further evaluate the selectivity of the sensor with the 5% ZrO_2_–ZnO nanocomposite, the response values of pure ZnO and 5% ZrO_2_–ZnO sensors were tested for 100 ppm of different gases, including isopropanol (CH_3_CHOHCH_3_), methanol (CH_3_OH), formaldehyde (HCHO), toluene (C_7_H_8_) and xylene (C_8_H_10_). As shown in Fig. 10, the 5% ZrO_2_–ZnO nanocomposite sensor had the highest response (172.46) to isopropanol. The sensitivities for other gases was <10. Therefore, the 5% ZrO_2_–ZnO nanocomposite sensor presents a good selectivity for isopropanol.

In addition, the prepared sensors were compared with other sensors, and the results are shown in Table 2. It can be found that the ZrO_2_–ZnO isopropanol sensor prepared in this work had the highest response when compared with previous ZnO gas sensors.

**Table 2:** Comparison of the gas sensing performance of our sensor and those reported in previous literature

Material	Temp. (°C)	Target gas	Conc. (ppm)	Response (*R*_a_/*R*_g_)	Ref.
Au–ZnO	300	Isopropanol	100	160	44
SnO_2_–ZnO	300	Isopropanol	500	98	45
CdS NP–ZnO PNs	320	Isopropanol	100	33	46
NiO–ZnO	280	Isopropanol	100	57	47
ZnO	320	Ethanol	100	3.3	48
Co–ZnO	300	Ethanol	100	90.71	49
Fe–ZnO nanostructure	370	Ethanol	100	45	50
ZrO_2_–ZnO	260	Isopropanol	100	172.46	This work

### 3.3 Gas sensing mechanisms

**Fig. 11:**
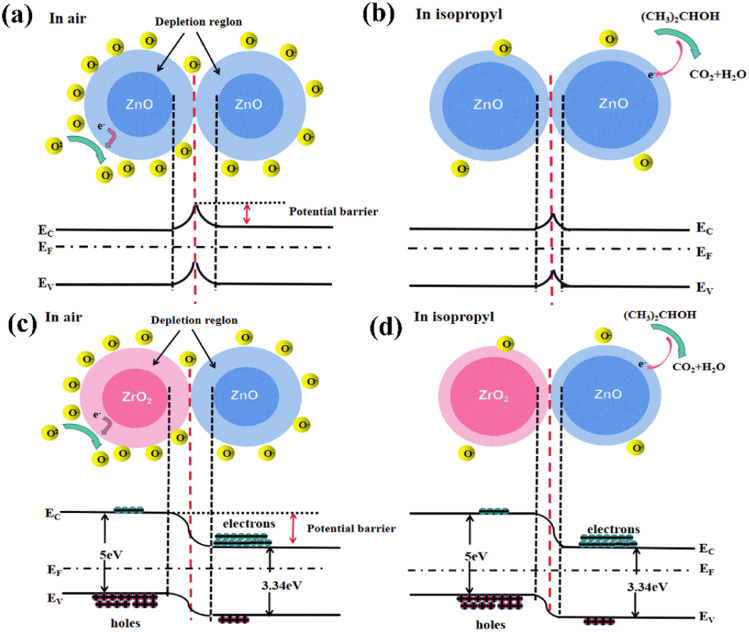
Sensing mechanism of ZrO_2_–ZnO composites in air and isopropanol atmosphere.

Generally, the gas-sensitive mechanism of n-type MOSs can be explained based on the electron depletion layer, which mainly involves three components: gas adsorption, electron transfer, and gas desorption.^7,31,50^ As shown in Fig. 11(a), the oxygen in the air is adsorbed onto the surface of the material when ZnO is exposed to air, which traps free electrons in the conduction band and forms oxygen ions. The process can be described by the following reactions:

1 O_2_(gas) → O_2_(ads)

2 O_2_(ads) + e^−^ → O_2_^−^(ads) (*T* < 100 °C)

3 O_2_^−^(ads) + e^−^ → 2O^−^(ads) (100 °C < *T* < 300 °C)

4 O^−^(ads) + e^−^ →O^2−^(ads) (*T* > 300 °C)

The loss of electrons on the oxide surface results in the formation of a depletion layer and an increase in the potential barrier, which, in turn, results in a higher resistance. When the sensor is exposed to isopropanol, the isopropanol reacts with the oxygen anions on the surface and releases electrons back into the material. The isopropanol is decomposed into oxygen and water, causing the surface depletion layer to decrease and the resistance to decrease; this process can be seen in Fig. 11(b) and described using the following reactions^51,52^:

5 (CH_3_)_2_CHOH_gas_ → (CH_3_)_2_CHOH_ads_

6 2(CH_3_)_2_CHOH_ads_ + 18O_ads_^−^ → 6CO_2_ + 8H_2_O + 18e^−^

7 (CH_3_)_2_CHOH_ads_ + 9O^2−^→ 3CO_2_ + 4H_2_O + 18e^−^

It has previously been reported that the response of gas-sensitive materials is related to the specific surface area. Generally, a large specific surface area produces more absorbed oxygen and the number of active sites involved in the redox reaction will increase,^43,48^ enhancing the response of the sensitive material. In contrast to pure ZnO, the 5% ZrO_2_–ZnO nanocomposite with a uniform rod-like structure has a larger specific surface area, which leads to a higher response for isopropanol. All ZrO_2_–ZnO materials showed a higher response than pure ZnO.

The catalytic effect of ZrO_2_ should also be considered. Tanabe reported that a methyl group of the absorbed isopropanol and a surface OH group of ZrO_2_ generate a hydrogen exchange reaction.^53^ ZrO_2_ has a role as an acid-based bifunctional catalyst in the activation of the methyl group. Therefore, ZrO_2_–ZnO samples show a higher response and a faster response time.

The ZrO_2_–ZnO heterojunction structure also contributes to the increase in the gas response. ZnO is a typical n-type semiconductor, and ZrO_2_ presents as a p-type semiconductor.^40^ As shown in Fig. 11(c), the ZrO_2_–ZnO composites form a p–n heterojunction structure. The electrons in the n-type ZnO and the holes in the p-type ZrO_2_ move in opposite directions to form built-in power plants, equalizing the Fermi energy levels and bending the energy band. A further increase in resistance is caused by the creation of heterojunctions. The sensor resistance of pure ZnO is 501.04 kΩ in air at 260 °C, while that of 5% ZrO_2_–ZnO reaches 97.65 MΩ under the same conditions. Consequently, ZrO_2_–ZnO sensor is exposed to reduced isopropanol, and the combination of the released electrons and holes in ZrO_2_ will lead to a narrowing of the depletion layer. The sensor resistance of ZrO_2_–ZnO decreases, and the response is further enhanced.

## 4. Conclusion

In this paper, pure ZnO and ZrO_2_–ZnO composites are prepared by a simple hydrothermal method and their structure and morphology is characterized. The morphology of pure ZnO is sheet-like, while the ZrO_2_–ZnO morphology presents as rod-like. The catalytic effects of ZrO_2_ may be the key factor in the formation of the rod-like structure. We investigated the gas-sensitive properties of the material to isopropanol. The sensor with 5% ZrO_2_–ZnO showed good gas-sensitive properties to isopropanol. Compared with pure ZnO, the 5% ZrO_2_–ZnO sensor achieved the highest response of 172.46 for 100 ppm isopropanol with an optimum operating temperature of 260 °C. The larger specific surface area, the catalytic effect of ZrO_2_ and the ZrO_2_–ZnO heterojunction structure are possible reasons for the increased gas response of ZnO materials. These results confirmed that doping with ZrO_2_ enhances the gas response of MOS materials and provides a new route toward developing gas-sensitive materials.

## Conflicts of interest

There are no conflicts to declare.

## Supplementary Material
